# Adaptive activation of EFNB2/EPHB4 axis promotes post-metastatic growth of colorectal cancer liver metastases by LDLR-mediated cholesterol uptake

**DOI:** 10.1038/s41388-022-02519-z

**Published:** 2022-11-14

**Authors:** Chunjie Xu, Lei Gu, Manzila Kuerbanjiang, Chunhui Jiang, Lipeng Hu, Ye Liu, Hanbing Xue, Jun Li, Zhigang Zhang, Qing Xu

**Affiliations:** 1 Department of Gastrointestinal Surgery, Renji Hospital, School of Medicine, Shanghai Jiao Tong University, 160 Pujian Road, Shanghai, China; 2 State Key Laboratory of Oncogenes and Related Genes, Shanghai Cancer Institute, Shanghai Jiao Tong University, Shanghai, PR China; 3 Division of Gastroenterology and Hepatology; Key Laboratory of Gastroenterology and Hepatology, Ministry of Health; Renji Hospital, School of Medicine, Shanghai Jiao Tong University; Shanghai Institute of Digestive Disease, 145 Middle Shandong Road, Shanghai, China

**Keywords:** Gastrointestinal cancer, Metastasis

## Abstract

The microenvironment of distant organ plays vital roles in regulating tumor metastases. However, little is known about the crosstalk between metastasized tumor cells and target organs. Herein, we found that EFNB2 expression was upregulated in liver metastases (LM) of colorectal cancer (CRC), but not in pulmonary metastases (PM) or primary CRC tumors. EFNB2 played a tumor-promoting role in CRC LM in vitro and in vivo. Through forward signaling, EFNB2-promoted CRC LM by interacting with the EPHB4 receptor. EFNB2/EPHB4 axis enhances LDLR-mediated cholesterol uptake in CRC LM. Subsequently, EFNB2/EPHB4 axis promotes LDLR transcription by regulating STAT3 phosphorylation. Blocking LDLR reversed the role of the EFNB2/EPHB4 axis in promoting CRC LM. Using clinical data, survival analysis revealed that the survival time of patients with CRC LM was decreased in patients with high EFNB2 expression, compared with low EFNB2 expression. Inhibition of the EFNB2/EPHB4 axis markedly prolonged the survival time of BALB/c nude mice with CRC LM with a high cholesterol diet. These findings revealed a key step in the regulation of cholesterol uptake by EFNB2/EPHB4 axis and its tumor-promoting role in CRC LM.

## Introduction

Colorectal cancer (CRC) is one of most common malignant tumors. According to the latest epidemiological report, CRC ranks third in both cancer incidence and mortality. The existence of distant metastases of CRC, primarily liver metastasis (LM) and pulmonary metastasis (PM), are the major independent factors indicating poor prognosis [1, 2].

Cancer metastasis is a complicated process, dependent upon invasion of tumor cells at the primary site, stage of the circulating tumor cells, metastatic colonization, and subsequent formation of metastases [3, 4]. Metastasized tumors show genetic alterations compared with primary tumors. Many genes involved in the process of cancer metastasis, but the pro-metastatic effect of the molecules produced by these genes not necessarily have target organ specificity. Emerging evidence indicates that different cell lines of breast cancer show completely different preferences for metastatic target organs [5]. Recent study also demonstrates very different characteristics of gene expression in metastasized tumors of different target organs in CRC [6]. Disseminated tumor cells express specific genes that can better adapt the microenvironment of target organs [7]. LM has the highest frequency of metastasis of CRC cells to distant organs [8, 9]. More than 50% of CRC patients suffer from LM during disease process. Despite the rapid development of diagnostic and therapeutic technology, fewer than 10% of CRC patients with LM live beyond 5 years [10]. Current therapeutic strategy for CRC LM has no liver metastases specifically. Therefore, the molecular mechanisms by which CRC cells grow in liver is crucial, and targeting such mechanisms provide novel horizons to develop accurate therapeutics against CRC LM.

Axon guidance factors are a series of important molecules in embryonic development, function to guide the growth of axons and establish neural circuitsact [11–13]. These molecules were divided into four subgroups, namely Semaphorins-Plexins, Netrins-DCC/UNCs, Slits-Robos and Ephrins-Ephs families. Recently, evidence has indicated an important effect of axon guidance factors on tumorigenesis [14]. Some axon guidance factors, including SLITs and SEMAs, affect the malignant behavior of tumor cells [15, 16]. However, the role and mechanism of axon guidance factors on liver metastases of CRC are still poorly understood.

In the present study, we explored whether is there a group of genes exhibiting altered expression in CRC LM due to adaptation to the liver environment. Using data analysis, we found that the expression of EFNB2 was upregulated in LM. and EFNB2/EPHB4 axis promoted LDLR-mediated cholesterol uptake, and eventually promoted the colonization and growth of CRC LM.

## Results

### Specific upregulation of EFNB2 in CRC LM contributes to post-metastatic growth

**Fig. 1:**
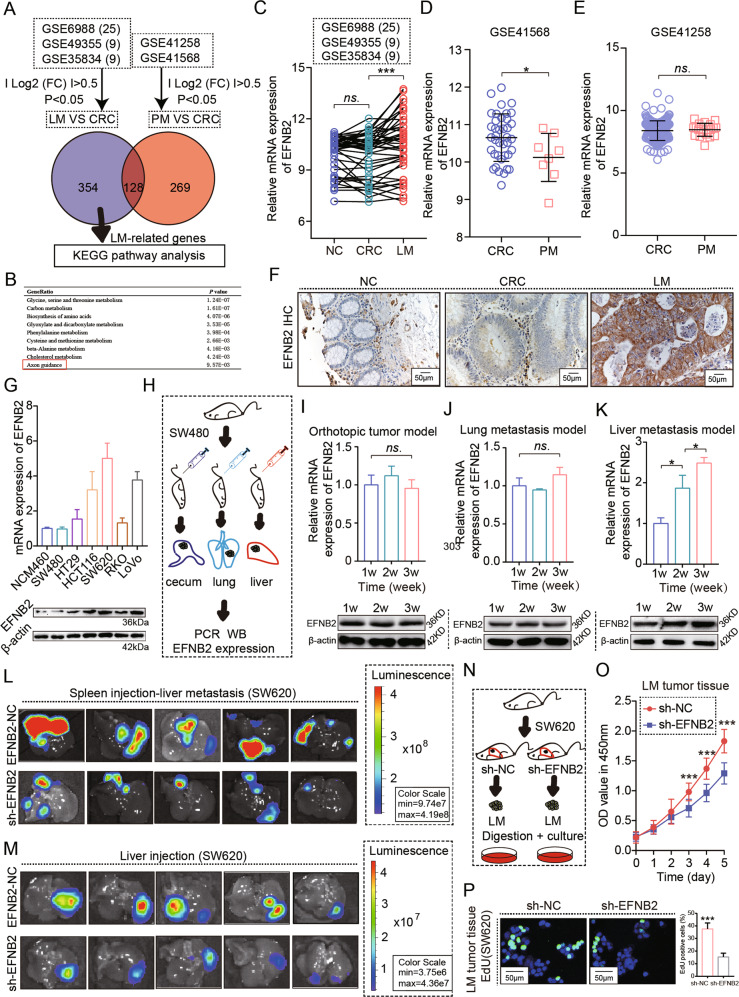
Increased expression of EFNB2 in CRC liver metastases contributes to post-metastatic growth. **A** Upregulated genes identified were GSE49355, GSE6988, and GSE35834 (CRC LM), and GSE41258 and GSE41568 (CRC PM). **B** KEGG pathway analysis of upregulated genes in CRC LM. **C** mRNA expression of EFNB2 in NC, CRC, and LM samples in GSE49355, GSE6988, and GSE35834. **D** mRNA expression of EFNB2 in CRC and PM samples in GSE41568. **E** mRNA expression of EFNB2 in CRC and PM samples in GSE41258. **F** Protein expression of EFNB2 in matched NC, CRC, LM tissues (*n* = 15 per group), Scale bar: 50 μm. **G** mRNA and protein expression of EFNB2 in NCM460 and six CRC cell lines. **H** Orthotopic tumor models, a liver metastasis model by spleen injection and a lung metastasis model by tail vein injection, were established by injecting SW480 cells into null mice. **I** mRNA and protein expression of EFNB2 in orthotopic tumor tissue at different time points (1 week, 2 weeks, and 3 weeks) (*n* = 6). **J** mRNA and protein expression of EFNB2 in lung metastasis tumor tissue at different time points (1 week, 2 weeks, and 3 weeks) (*n* = 6). **K** mRNA and protein expression of EFNB2 in liver metastasis tumor tissue at different time points (1 week, 2 weeks, and 3 weeks) (*n* = 6). **L** Imaging of animal model of liver metastasis by spleen injection with sh-EFNB2 or sh-NC SW620^Luc^ cells (*n* = 6). Scale color bar: 9.74 × 10^7^–4.19 × 10^8^. **M** Imaging of animal intrahepatic model by liver injection with sh-EFNB2 or sh-NC SW620^Luc^ cells (*n* = 5 per group). Scale color bar: 3.75 × 10^6^–4.36 × 10^7^. **N** LM tumor tissues from sh-EFNB2 or sh-NC mice were digested and LM cells were cultured. **O** The viability of SW620 cells from LM tumor tissues in sh-EFNB2 and sh-NC groups, as analyzed using CCK-8 assays. **P** Proliferation of SW620 cells from LM tumor tissues in sh-EFNB2 and sh-NC groups, as analyzed using EdU assays. Scale bar: 50 μm. All experiments were performed in triplicate. Measurement data were presented as the mean ± SD. Student’s *t*-tests were used for statistical analysis. ns. represents no statistical difference; *p < 0.05; ***p < 0.001.

To characterize the gene sets involved specifically in CRC metastasis to the liver, not to the lung, we first analyzed the GSE41258 (CRC LM) and GSE6988 (CRC PM) datasets, and identified genes specially upregulated in CRC LM, but not in CRC PM. Of these, EFNB2, an axon guidance factor, aroused our interest (Fig. 1A–E). According to IHC staining, EFNB2 was significantly upregulated in paired CRC LM tissues, but not CRC tissues, compared with adjacent paired noncancerous tissues (Fig. 1F). To further confirm the expression pattern of EFNB2, we established three types of CRC models (Fig. 1G, H), including an orthotopic tumor model, an LM model by spleen injection, and a PM model by tail vein injection. EFNB2 expression showed no obvious changes in tumor tissues at different time points in the orthotopic tumor model or the PM model (Fig. 1I, J), but was gradually upregulated in tumor tissues at different time points in the LM model (Fig. 1K). These results indicated a probable association of EFNB2 upregulation with CRC LM.

Next, migration assay showed EFNB2 knockdown rarely affected migration ability (Fig. S1A, B). To confirm the effect of EFNB2 on CRC LM, we injected sh-NC or sh-EFNB2 SW620 cells into the spleen of nude mice to establish the LM model and found that EFNB2 knockdown reduced the tumor burden of CRC LM (Figs. 1L, S1C). Then, we injected sh-EFNB2 or sh-NC SW620 cells into the liver of nude mice, and found that EFNB2 knockdown significantly inhibited the growth of CRC tumor tissue in the liver (Figs. 1M and S1D). LM tumor tissues were digested and cultured (Fig. 1N), and CCK-8 and EdU assays showed EFNB2 knockdown significantly inhibited the proliferation ability of CRC LM cells in vitro (Fig. 1O, P). These data demonstrated that EFNB2-promoted colonization and growth of CRC LM in a liver-dependent manner.

### EFNB2 promotes post-metastatic growth of CRC LM via forward signaling

**Fig. 2:**
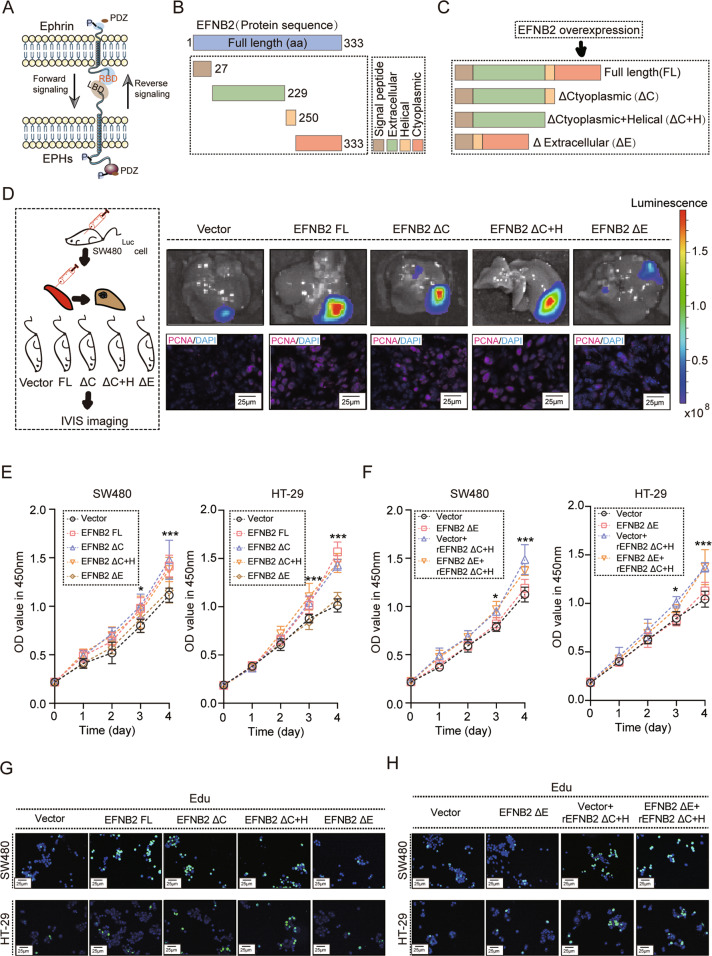
EFNB2 forward signaling, not reverse signaling, promotes post-metastatic growth of CRC LM. **A** Schematic diagram of signal conduction direction. **B** Protein structure of EFNB2. **C** Construction of four types of EFNB2 overexpression plasmids, including EFNB2 full length (FL), EFNB2 ΔC, EFNB2 ΔC + H, and EFNB2 Δ*E*. **D** Liver metastasis model by spleen injection with vector, EFNB2 FL, EFNB2 ΔC, EFNB2 ΔC + H, and EFNB2 Δ*E* SW480^Luc^ cells (*n* = 6 per group) and animal imaging to monitor tumor growth and staining PCNA in LM tumor tissues (Purple: PCNA, Blue: DAPI). Scale bar: 25 μm. **E** Viability of SW480 and HT29 cells transfected with vector, EFNB2 FL, EFNB2 ΔC, EFNB2 ΔC + H, and EFNB2 ΔE, as analyzed using CCK-8 assays. **F** Viability of SW480 and HT29 cells transfected with vector, EFNB2 ΔE, Vector + rEFNB2 ΔC + H, and EFNB2 ΔE + rEFNB2 ΔC + H, as analyzed using CCK-8 assays. **G** EdU assays of SW480 and HT29 cells transfected with vector, EFNB2 FL, EFNB2 ΔC, EFNB2 ΔC + H, and EFNB2 ΔE. **H** EdU assays of SW480 and HT29 cells transfected with vector, EFNB2 ΔE, Vector + rEFNB2 ΔC + H, and EFNB2 ΔE + rEFNB2 ΔC + H. Scale bar: 25 μm. All experiments were performed in triplicate. Measurement data are presented as the mean ± SD. Student’s *t*-tests were used for statistical analysis. ns. represents no statistical difference; *p < 0.05; ***p < 0.001.

Ephrins-Ephs interaction is complex. Eph proteins behave as classical receptors, and Ephrins as ligands, which is termed Ephrins-Ephs forward signaling. Eph proteins behave as ligands signaling to Ephrin, known as reverse signaling (Fig. 2A). To explore the main pattern of signal transduction, we established a plasmid with ectopic expression of a C-terminal truncated form of EFNB2 (ΔC EFNB2 and ΔC + H EFNB2), which disrupted reverse signaling, and an N-terminal truncated form of EFNB2 (ΔE EFNB2), which disrupted forward signaling (Fig. 2B, C). SW480 cells transfected with EFNB2 full length (EFNB2 FL) exhibited more severe metastatic tumor burden than transfected with empty vector. Disruption of reverse signaling by transfection with ΔC EFNB2 and ΔC + H EFNB2 showed a comparable metastatic tumor burden with EFNB2 FL, indicating that reverse signaling of EFNB2 was not involve in promoting post-metastatic growth of CRC LM (Figs. 2D and S2A). In contrast, disruption of forward signaling by transfection with ΔE EFNB2 did not promote post-metastatic growth of CRC LM compared with empty vector transfection (Figs. 2D and S2A). In vitro experiments showed similar results (Figs. 2E, G and S2B). Purified protein from ΔC + H EFNB2 CRC cells significantly increased the viability of CRC cells (Figs. 2F, H and S2C). Therefore, EFNB2 forward signaling, not reverse signaling, was involved in post-metastatic growth of CRC LM.

### EFNB2/EPHB4 axis enhanced post-metastatic growth of CRC LM

**Fig. 3:**
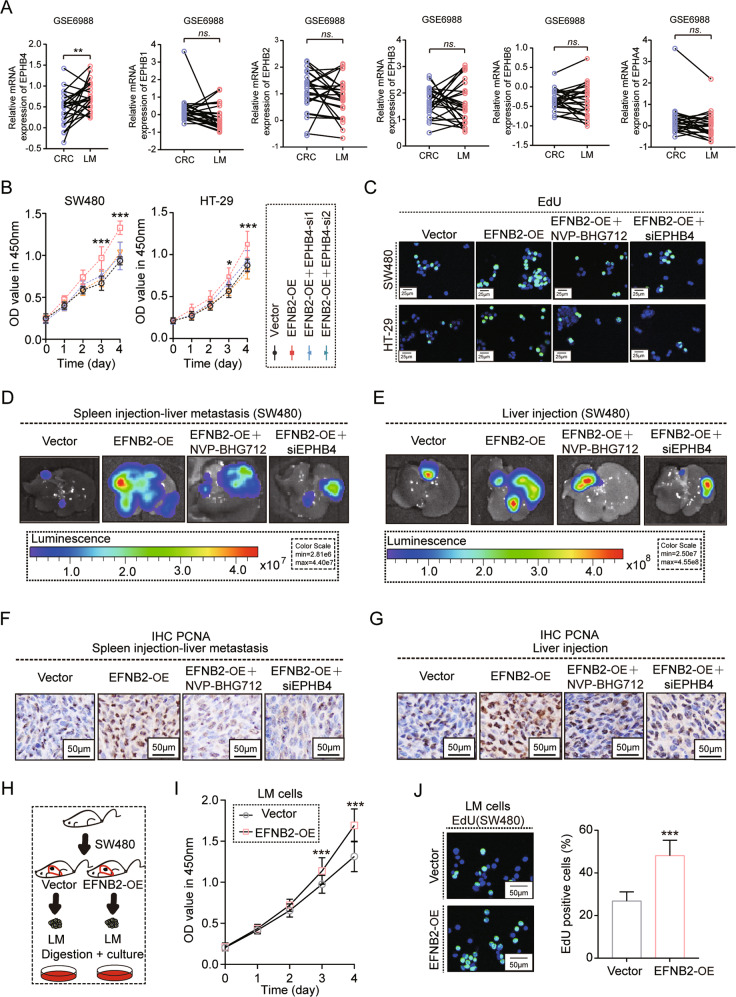
The EFNB2-EPHB4 axis enhanced post-metastatic growth of CRC LM. A mRNA expression of EPHB4 and other EPHs in CRC and LM samples in GSE6988. **B** Viability of SW480 and HT29 cells transfected with vector, EFNB2-OE, and EFNB2-OE + siEPHB4, as analyzed using CCK-8 assays. **C** EdU assays of SW480 and HT29 cells transfected with vector, EFNB2-OE, EFNB2-OE + siEPHB4, and EFNB2-OE + NVP-BHG712. Scale bar: 25 μm. **D** Liver metastasis model created by spleen injection with SW480^luc^ cells transfected with vector, EFNB2-OE, EFNB2-OE + siEPHB4, and EFNB2-OE + NVP-BHG712 (*n* = 6 per group), and animal imaging to monitor tumor growth. Scale color bar: 2.81 × 10^6^–4.40 × 10^7^. **E** Liver infection model with SW480^luc^ cells transfected with vector, EFNB2-OE, EFNB2-OE + siEPHB4, and EFNB2-OE + NVP-BHG712 (*n* = 6 per group), and animal imaging to monitor tumor growth. Scale color bar: 2.50 × 10^7^–4.55 × 10^8^. **F** PCNA staining by IHC in LM tumor tissues (vector, EFNB2-OE, EFNB2-OE + siEPHB4, and EFNB2-OE + NVP-BHG712). Scale bar: 50 μm. **G** PCNA staining by IHC in tumor tissues (vector, EFNB2-OE, EFNB2-OE + siEPHB4, and EFNB2-OE + NVP-BHG712). Scale bar: 50 μm. **H** LM tumor tissues from vector or EFNB2-OE mice were digested and LM cells were cultured. **I** The viability of SW480 cells from LM tumor tissues in the vector and EFNB2-OE groups, as analyzed using CCK-8 assays. **J** The proliferation of SW480 cells from LM tumor tissues in the vector and EFNB2-OE groups, as analyzed using EdU assays. Scale bar: 50 μm. All experiments were performed in triplicate. Measurement data are presented as the mean ± SD. Student’s *t*-tests were used for statistical analysis. ns. represents no statistical difference; *p < 0.05; **p < 0.01; ***p < 0.001.

Generally, EFNB2 interacts with EPH receptors, which in turn transmit downstream signals. First, we analyzed the mRNA expression of EPH receptors in the CRC LM from GSE6988. EPHB4, but not other EPHs, was more highly expressed in LM than in paired CRC samples (Fig. 3A). In vitro experiments showed that EFNB2 promoted the viability of CRC cells via EPHB4, but not the other EPH receptors (EPHB1, EPHB2, EPHB3, EPHB6, and EPHA4) (Figs. 3B and S3A–S3F). EPHB4, as a receptor tyrosine kinase, transmits extracellular signals by phosphorylating its phosphorylation sites. NVP-BHG712 was used to specifically block the EPHB4 receptor by inhibiting its phosphorylation site. EdU assays showed that blocking EPHB4 severely inhibited the proliferation of CRC cells by EFNB2 overexpression (Figs. 3C and S3G). To further confirm the effect of the EFNB2/EPHB4 axis on CRC LM, we injected EFNB2-OE or empty vector SW480^luc^ cells into the spleen of nude mice to establish the LM model, or directly injected them into the liver. EFNB2-promoted tumor growth in the LM and liver injection models was significantly reduced after the inhibition of EPHB4 by siRNA and NVP-BHG712 (Fig. 3D, E). PCNA staining also showed that blocking EPHB4 reversed EFNB2-promoted cell proliferation in the LM and liver injection models (Figs. 3F, G and S3H, I). LM tumor tissues were digested and cultured (Fig. 3H), CCK-8 and EdU assays showed that EFNB2 overexpression significantly promoted the proliferation ability of CRC LM cells in vitro (Fig. 3I, J). These data demonstrated that EFNB2 promotes the LM of CRC via interacting with the EPHB4 receptor.

### The EFNB2/EPHB4 axis promotes cholesterol uptake via regulating LDLR expression

**Fig. 4:**
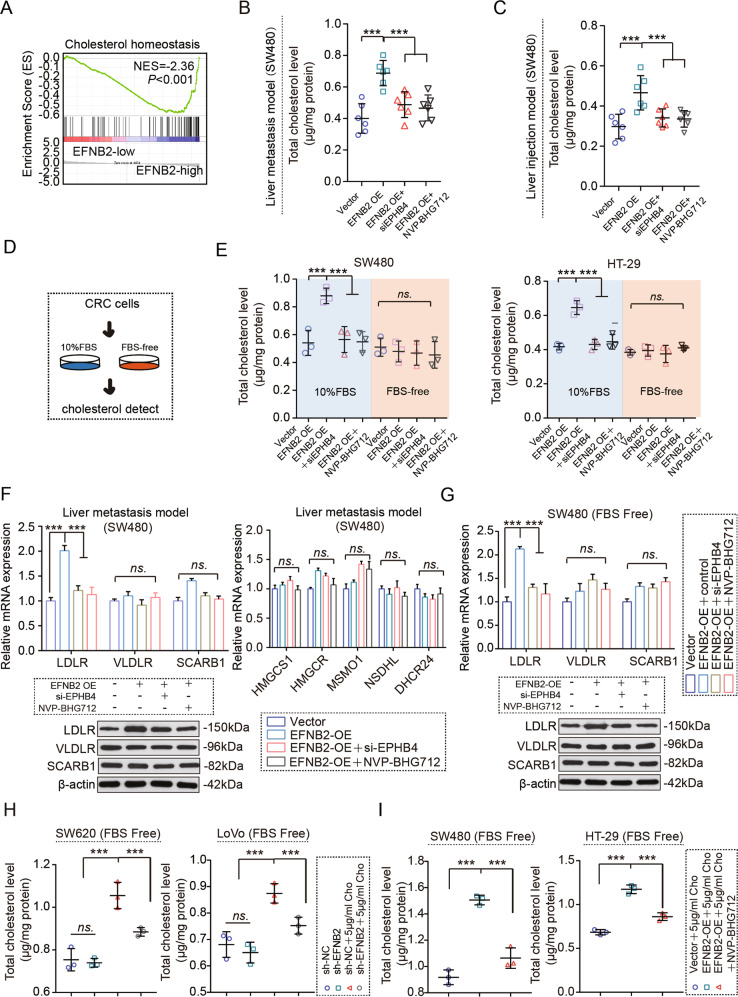
The EFNB2/EPHB4 axis promotes cholesterol uptake by regulating the expression of LDLR. **A** GSEA analysis of EFNB2 expression in CRC, as evaluated using GSE6988. A total of 24 CRC LM samples were divided into two groups, including a high EFNB2 expression group (12 samples) and a low EFNB2 expression group (12 samples). **B** Total cholesterol levels were measured in the CRC LM tumor tissues, including the vector, EFNB2-OE, EFNB2-OE + siEPHB4, and EFNB2-OE + NVP-BHG712 groups (*n* = 6 per group). **C** Total cholesterol levels in the tumor tissues of the liver injection model, including the vector, EFNB2-OE, EFNB2-OE + siEPHB4, and EFNB2-OE + NVP-BHG712 groups (*n* = 6 per group). **D** Cholesterol levels were measured in CRC cells. **E** Cholesterol levels were measured in SW480 and HT29 cells, including the vector, EFNB2-OE and EFNB2-OE + siEPHB4, EFNB2-OE + NVP-BHG712 groups, with or without 10% FBS treatment. **F** Expression of cholesterol uptake related genes (LDLR, VLDLR, and SCARB1), and cholesterol synthesis related genes (HMGCS1, HMGCR, NSDHL, MSMO1, and DHCR24) in CRC LM tumor tissues, including the vector, EFNB2-OE, EFNB2-OE + siEPHB4, and EFNB2-OE + NVP-BHG712 groups (*n* = 6 per group). **G** Expression of cholesterol uptake related genes (LDLR, VLDLR, and SCARB1), and cholesterol synthesis related genes (HMGCS1, HMGCR, NSDHL, MSMO1, and DHCR24) in SW480 cells in FBS-free culture, including the vector, EFNB2-OE, EFNB2-OE + siEPHB4, and EFNB2-OE + NVP-BHG712 groups. **H** Total cholesterol levels in SW620 and LoVo cells in FBS-free culture, including sh-NC, sh-EFNB2, sh-NC + 5 μg/ml cholesterol (Cho), and sh-EFNB2 + 5 μg/ml Cho. **I** Total cholesterol levels in SW480 and HT29 cells in FBS-free culture, including vector + 5 μg/ml Cho, EFNB2-OE + 5 μg/ml Cho, and EFNB2-OE + 5 μg/ml Cho + NVP-BHG712. All experiments were performed in triplicate. Measurement data are presented as the mean ± SD. Student’s *t*-tests were used for statistical analysis. ns. indicated no statistical difference; ***p < 0.001.

To further determine the underlying mechanism by which the EFNB2/EPHB4 axis contributes to CRC LM, we conducted a Gene Set Enrichment Analysis (GSEA) analysis based on EFNB2 expression in the CRC LM samples. The gene sets from the high EFNB2 group were enriched in cholesterol homeostasis (Fig. 4A), suggesting a potential link between the EFNB2/EPHB4 axis and cholesterol in CRC LM. The total cholesterol level was higher in LM tissues with high EFNB2 expression, compared with low EFNB2 expression (Table S1). In tissues from both the CRC LM and liver injection models, the total cholesterol level was increased in EFNB2-OE metastatic cells (Fig. 4B, C) and decreased in sh-EFNB2 metastatic cells (Fig. S4A). Blocking of EPHB4 by siRNA or NVP-BHG712 reversed the cholesterol level elevated by EFNB2 overexpression in the metastasized CRC cells (Fig. 4B, C). Analysis of the CRC LM (GSE6988, GSE35834 and GSE49355), compared with CRC primary tumor, indicated that the expression of genes related to cholesterol synthesis (HMGCS1, HMGCR, MSMO1 and DHCR24) was inhibited, and that of cholesterol uptake related genes (LDLR, VLDLR and SCARB1) was upregulated in CRC LM (Fig. S4B–H). Liver is the central organ of cholesterol homeostasis, and has a higher level of cholesterol than the colorectum or lung [17]. In vitro experiments showed that the EFNB2/EPHB4 axis increased the total cholesterol level in SW480 and HT29 cells cultured with 10% FBS. However, this effect was almost eliminated in CRC cells cultured with FBS-free medium (Fig. 4D, E). We next analyzed the correlation between EFNB2 and the expression of genes related to cholesterol synthesis and uptake in the CRC LM, using the GSE6988 dataset. The mRNA expression of EFNB2 was significantly positively correlated with cholesterol uptake related genes (LDLR, VLDLR, and SCARB1) (Fig. S5A), but not cholesterol synthesis related genes (HMGCS1, HMGCR, NSDHL, MSMO1, and DHCR24) (Fig. S5B). In the CRC LM model, LDLR was upregulated in EFNB2-OE metastatic cells (Fig. 4F) and downregulated in sh-EFNB2 metastatic cells (Fig. S5C). The EFNB2/EPHB4 axis did not regulate the gene expression of cholesterol synthesis (Figs. 4F and S5C). Blocking of EPHB4 by siRNA and NVP-BHG712 reversed the elevated expression of LDLR caused by EFNB2 overexpression (Fig. 4F). In in vitro experiments, after restricting the exogenous cholesterol by FBS-free medium, the EFNB2/EPHB4 axis still promoted LDLR expression (Figs. 4G and S5D). With exogenous cholesterol treatment, EFNB2 knockdown reduced the cholesterol level (Fig. 4H) and blocking EPHB4 inhibited the elevated levels of cholesterol caused by EFNB2 overexpression in FBS-free cultured CRC cells (Fig. 4I). Taken together, the EFNB2/EPHB4 axis promotes cholesterol uptake via the regulation of LDLR expression in CRC LM.

### The EFNB2/EPHB4 axis promotes LDLR transcription by STAT3 activation

**Fig. 5:**
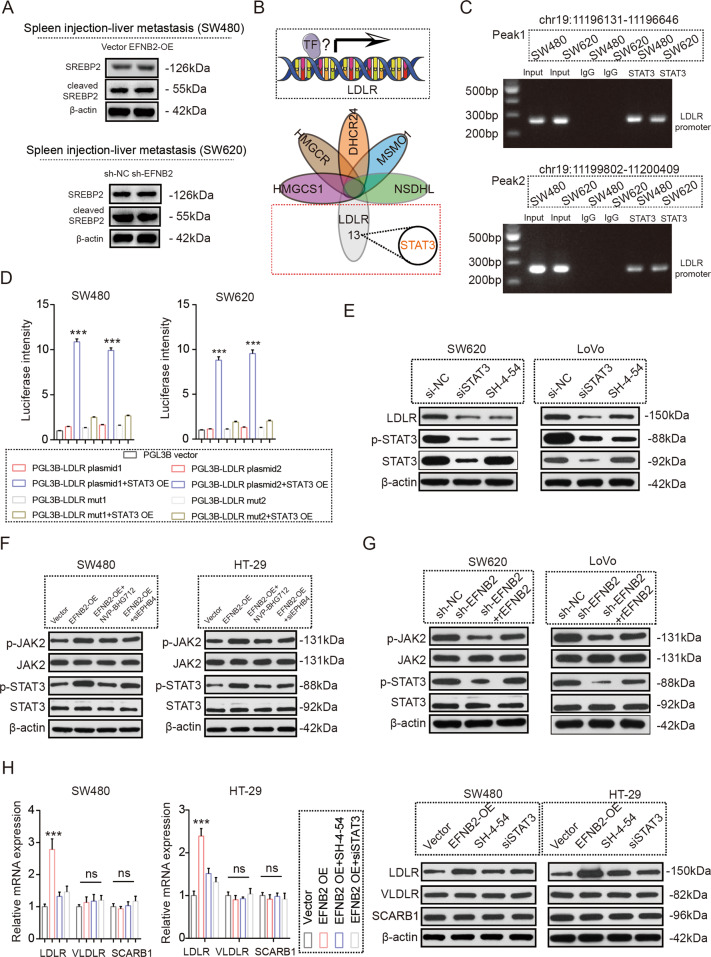
The EFNB2/EPHB4 axis promotes LDLR transcription by STAT3. **A** Protein expression of total SREBP2 and activated SREBP2 in CRC LM tissues transfected with vector and EFNB2-OE (*n* = 6). **B** Transcription factor analysis of LDLR. **C** STAT3 binding to the promoter region of LDLR in SW480 and SW620 cells, as assessed using ChIP assay. **D** Role of STAT3 in promoting LDLR transcription, according to a dual luciferase report assay. **E** Protein expression of LDLR, STAT3, and p-STAT3 in SW620 and LoVo cells, including si-NC, siSTAT3, and SH-4-54 groups. **F** Protein expression of JAK2, p-JAK2, STAT3, and p-STAT3 in SW480 and HT29 cells, including the vector, EFNB2-OE, EFNB2-OE + NVP-BHG712, and EFNB2-OE + siEPHB4 groups. **G** Protein expression of JAK2, p-JAK2, STAT3, and p-STAT3 in SW620 and LoVo cells, including the sh-NC, sh-EFNB2, and sh-EFNB2 + rEFNB2 groups. **H** mRNA and protein expression of LDLR, VLDLR, and SCARB1 in SW480 and HT29 cells, including the vector, EFNB2-OE, EFNB2-OE + SH-4-54, and EFNB2-OE + siSTAT3 groups. All experiments were performed in triplicate. Measurement data are presented as the mean ± SD. Student’s *t*-tests were used for statistical analysis. ns. indicates no statistical difference; ***p < 0.001.

Next, we explored the underlying mechanism by which the EFNB2/EPHB4 axis regulates LDLR expression. A recent study found that Sterol-Regulatory Element-Binding Protein 2 (SREBP2) is a vital transcription factor (TF) in cholesterol homeostasis [18]. However, the EFNB2/EPHB4 axis did not affect the expression and activation of SREBP2 (Fig. 5A). Subsequently, we predicted the potential TFs of LDLR and related genes of cholesterol synthesis, of which, Signal Transducer And Activator Of Transcription 3 (STAT3) is predicted to bind to the promoter region of LDLR, but not related genes involved in cholesterol synthesis (Fig. 5B). Subsequently, experiments showed that STAT3 bound to the promoter region of LDLR and activated its transcription (Fig. 5C, D). Mutation of the binding site of LDLR abolished the transcriptional activation of LDLR by STAT3 (Fig. 5D). STAT3 silencing by siRNA or a specific inhibitor (SH-4-54) downregulated LDLR expression in CRC cells (Fig. 5E). STATs could be phosphorylated by the receptor associated kinases, trans-located to the nucleus in a dimer form, and then activate the transcription of target genes. We also found that the EFNB2/EPHB4 axis promoted JAK2/STAT3 phosphorylation (Fig. 5F, G). STAT3 inhibition reversed LDLR upregulation by the EFNB2/EPHB4 axis (Fig. 5H). These results showed that the EFNB2/EPHB4 axis promotes LDLR expression by regulating STAT3 phosphorylation.

### LDLR mediates the promoting role of EFNB2/EPHB4 axis in the tumor growth of CRC LM

**Fig. 6:**
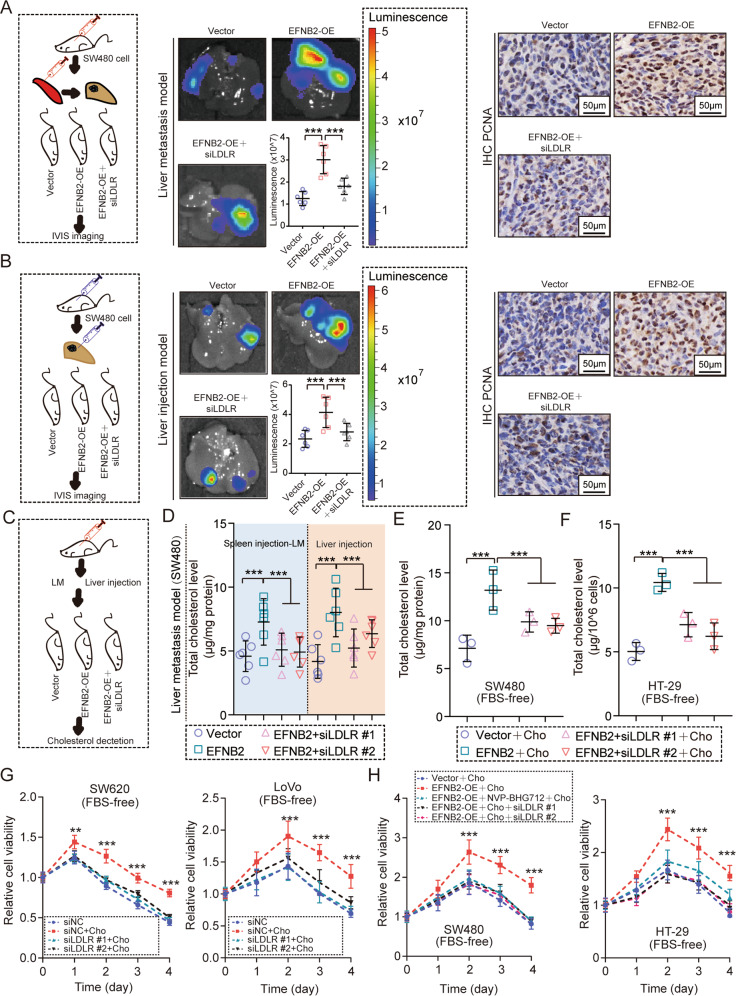
LDLR mediates the promoting role of the EFNB2/EPHB4 axis in CRC LM. **A** LM model created by spleen injection with SW480^luc^ cells transfected with vector, EFNB2-OE, EFNB2-OE + siLDLR #1, or EFNB2-OE + siLDLR #2 (*n* = 6 per group) and animal imaging to monitor tumor growth and PCNA staining by IHC in tumor tissues. Scale bar: 50μm. **B** Liver injection model created by spleen injection with SW480^luc^ cells transfected with vector, EFNB2-OE, EFNB2-OE + siLDLR #1, or EFNB2-OE + siLDLR #2 (*n* = 6 per group) and animal imaging to monitor tumor growth and PCNA staining by IHC in tumor tissues. Scale bar: 50 μm. **C** Cholesterol detection in an LM model transfected with vector, EFNB2-OE, EFNB2-OE + siLDLR #1, or EFNB2-OE + siLDLR #2 (*n* = 6 per group). **D** Total cholesterol levels in tumor tissues in an LM model and a liver injection model transfected with vector, EFNB2-OE, EFNB2-OE + siLDLR #1, or EFNB2-OE + siLDLR #2 (*n* = 6 per group). **E** Total cholesterol levels in SW480 CRC cells in FBS-free and cholesterol culture (5 μg/ml Cho), including vector, EFNB2-OE, EFNB2-OE + siLDLR #1, and EFNB2-OE + siLDLR #2 groups. **F** Total cholesterol levels in HT29 CRC cells in FBS-free and cholesterol culture (5 μg/ml Cho), including vector, EFNB2-OE, EFNB2-OE + siLDLR #1, and EFNB2-OE + siLDLR #2 groups. **G** The viability of SW620 and LoVo cells in FBS-free culture, including the siNC, siNC + 5 μg/ml Cho, siLDLR #1 + 5 μg/ml Cho, and siLDLR #2 + 5 μg/ml Cho groups, as analyzed using CCK-8 assays. **H** Viability of SW480 and HT29 cells in FBS-free culture, including vector + 5 μg/ml Cho, EFNB2-OE + 5 μg/ml Cho, EFNB2-OE + NVP-BHG712 + 5 μg/ml Cho, EFNB2-OE + siLDLR #1 + 5 μg/ml Cho, and EFNB2-OE + siLDLR #2 + 5 μg/ml Cho groups, as analyzed using CCK-8 assays. All experiments were performed in triplicate. Measurement data are presented as the mean ± SD. Student’s *t*-tests were used for statistical analysis. ns. indicates no statistical difference; **p < 0.01, ***p < 0.001.

As mentioned above, the EFNB2/EPHB4 axis transcriptionally regulated LDLR expression. However, whether LDLR is involved in the promoting effect of the EFNB2/EPHB4 axis on CRC LM was still unknown. In vivo experiments showed that LDLR inhibition by siRNA blocked the promoting role of EFNB2 overexpression in liver metastatic tumors of CRC (Fig. 6A, B). Inhibition of LDLR reversed the upregulation of the expression of PCNA caused by EFNB2 overexpression (Fig. 6A, B). Although the EFNB2/EPHB4 axis contributes to CRC LM by regulating LDLR, whether cholesterol uptake by LDLR participates in EFNB2/EPHB4 axis-derived CRC LM was still unclear. In vivo and in vitro experiments indicated that blocking LDLR reversed the elevation of cholesterol by the EFNB2/EPHB4 axis (Fig. 6C–F). Treatment with exogenous cholesterol showed an obvious pro-survival role in CRC cells cultured in FBS-free medium, and silencing LDLR completely inhibited this effect (Fig. 6G). Blocking the EFNB2/EPHB4 axis also inhibited its pro-survival role in CRC cells under cholesterol treatment. Restricting cholesterol intake by inhibiting LDLR attenuated the pro-survival role in CRC cells with EFNB2 overexpression (Fig. 6H). Thus, the EFNB2/EPHB4 axis promoted the growth of CRC LM via LDLR-mediated cholesterol uptake.

### EFNB2/EPHB4 axis is a promising therapeutic target for CRC LM

**Fig. 7:**
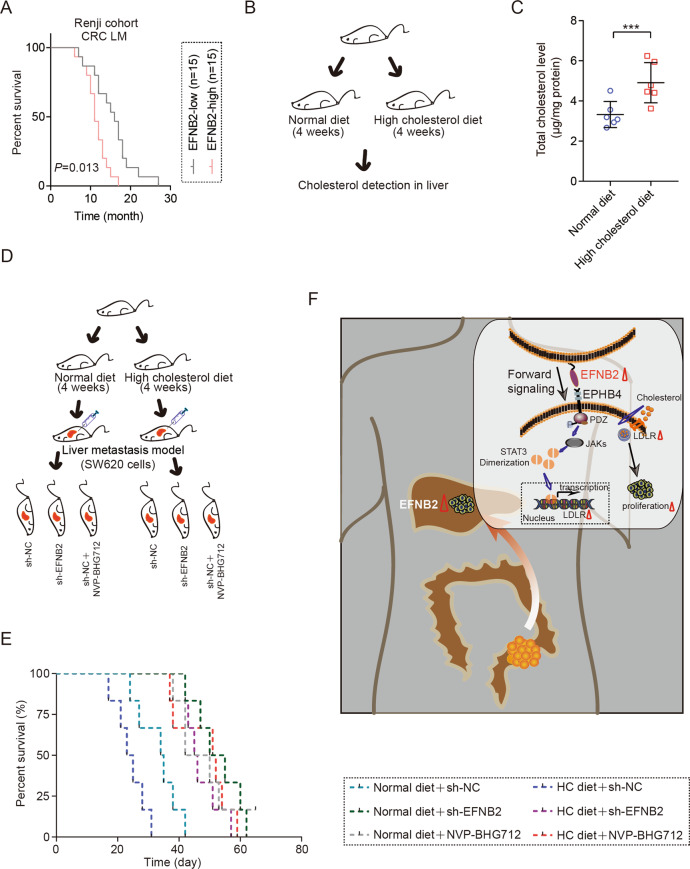
The EFNB2/EPHB4 axis is a therapeutic target for CRC LM. **A** Overall survival analysis of patients with CRC LM, based on the protein expression of EFNB2 (*n* = 30). **B** A high cholesterol model was established by providing a high cholesterol diet to BALB/c nude mice. **C** Total cholesterol levels in the liver tissues of mice with high cholesterol and normal control feeding (*n* = 6 per group). **D** CRC LM models were established using SW620 cells in mice with high cholesterol and normal control feeding, including the sh-NC, sh-EFNB2, and sh-NC + NVP-BHG712 groups (*n* = 6 per group). **E** Overall survival analysis of CRC LM models in mice with high cholesterol and normal control feeding, including the sh-NC, sh-EFNB2, and sh-NC + NVP-BHG712 groups (*n* = 6 per group). **F** Adaptive activation of the EFNB2/EPHB4 axis promoted CRC LM by LDLR-mediated cholesterol uptake. All experiments were performed in triplicate. Measurement data are presented as the mean ± SD. Student’s *t*-tests were used for statistical analysis. ***p < 0.001.

Survival analysis based on the expression level of EFNB2 revealed a significant decrease in the survival time of patients with CRC LM who had high EFNB2 expression, compared with those with low EFNB2 expression in LM tissues (Fig. 7A). The liver is the central organ of cholesterol synthesis and metabolism. In our study, by establishing a mouse model with a high cholesterol level in BALB/c nude mice, we found the cholesterol level was higher in the liver of mice with a high cholesterol diet than in mice with a normal diet (Fig. 7B, C). In the present study, we found aberrant activation of the EFNB2/EPHB4 axis enhanced LDLR-mediated cholesterol uptake. Given these results, we explored whether targeting the EFNB2/EPHB4 axis could have a beneficial effect on the prognosis of patients with CRC LM. In the LM model (Fig. 7D), we found that mice with a high cholesterol diet had a lower survival time than mice with a normal diet (Fig. 7E). Blocking the EFNB2/EPHB4 axis greatly improved the survival time of mice burdened with CRC LM (Fig. 7E). Blocking the EFNB2/EPHB4 axis almost eliminated the effect of a high cholesterol diet in the mice with CRC LM, indicating the importance of the EFNB2/EPHB4 axis in regulating cholesterol intake (Fig. 7E). In summary, once transferred to the liver, abnormal activation of the EFNB2/EPHB4 axis in metastasized CRC cells took up and utilized cholesterol from the outside environment, which promoted the colonization and growth of metastasized cells (Fig. 7F).

## Discussion

Due to the difference in structure and metabolic patterns of different organs, metastatic tumor cells show quite different expression profiles in different environments [6], indicating that tumor cells are necessary to adapt the microenvironment of metastatic organs via genetic alteration. In this article, we found that the expression of EFNB2 was upregulated in CRC LM, but not in primary CRC tumors or PM. EFNB2 was found to contribute to the post-metastatic growth of CRC LM. These data again indicated a close association between the upregulated expression of EFNB2 in CRC LM and tumor growth in the liver.

Ephrins are divided into two subgroups, Ephrin A and Ephrin B. Ephrins-EPHs signaling is extremely complex, and classified as classic ligand-to-receptor pattern (forward signaling), receptor-to-ligand pattern (transverse signaling), and bidirectional signaling [19]. Our previous study found that: [1] EFNB2 overexpression significantly promoted post-metastatic growth of CRC LM; [2] blocking EFNB2 forward signaling, but not reverse signaling, inhibited the promoting effect of EFNB2 overexpression on CRC LM. These results suggest that EFNB2 may promote the post-metastatic growth of CRC LM through forward signaling. EPHs are classical receptors of Ephrins, and are also divided into EPHA and EPHB receptors. Ordinarily, the ligands belonging to Ephrin A combine with EPHA receptors and those of Ephrin B combine with EPHB receptors. Ephrin B can also combine with EPHA4 [11]. EFNB2 plays a tumor-promoting role in pancreatic ductal adenocarcinoma, breast cancer, and glioblastoma [20–22]. A recent study showed that blocking EFNB2/EPHB4 signaling increased the response to cetuximab-radiation therapy in head and neck cancers [23]. However, the role of EFNB2/EPHs signaling in CRC metastasis was still unknown. We found that in CRC LM, EFNB2-promoted cell proliferation by interacting with EPHB4. Blocking EPHB4 eliminated the promoting effect of EFNB2 on CRC LM.

The liver is the central organ of cholesterol metabolism [24]. Approximately 80% of cholesterol in the human body is synthesized by hepatocyte, and metabolized into steroid hormones and bile acids, producing a high cholesterol microenvironment in the liver [25]. Cholesterol is an essential component of cell and organelle membranes [26]. The rapid proliferation of cancer cells requires the accelerated synthesis of cell and organelle membranes, and aberrant cholesterol metabolism therefore plays a vital role in tumor growth [27, 28]. We found that the EFNB2/EPHB4 axis increased total cholesterol levels in CRC LM. Intracellular cholesterol mainly depends on synthesis and extracellular uptake [29]. Bioinformatics analysis and in vivo and vitro modeling indicated that the EFNB2/EPHB4 axis upregulated the expression of LDLR, but not that of the synthetic genes of cholesterol in CRC LM. LDLR is the most important receptor for cholesterol uptake [30]. By binding with LDLR, the extracellular lipoprotein-cholesterol complex enters cells via endocytosis, and the cholesterol ester is hydrolyzed into free cholesterol by lysosomal enzymes, thus playing its physiological role [31]. In the present study, eliminating exogenous cholesterol inhibited the effect of the EFNB2/EPHB4 axis on regulating cholesterol levels in CRC cells cultured with FBS-free medium, and adding exogenous cholesterol enhanced the effect.

Sterol-regulatory element-binding protein 2 (SREBP2) is an intracellular cholesterol sensor located in the endoplasmic reticulum, which regulates intracellular cholesterol through the Insig-SREBP-SCAP pathway [32]. However, our data showed that the EFNB2/EPHB4 axis did not affect the expression or activation of SREBP2 in CRC LM. Using data analysis and experimental verification, we found that STAT3 had transcriptional activity toward LDLR. Once phosphorylated, STAT3 polymerizes into a homologous or heterodimer form of activated transcription activator, enters into the nucleus, and binds to specific sites of the target gene promoters to activate transcription [33]. The Eph receptor family, as tyrosine kinase receptors, also plays a signaling role through the phosphorylation of downstream kinases. EPHB2 promotes angiogenesis in tumor cells by introducing STAT3 phosphate into the nucleus [34]. EPHA4 has also been shown to phosphorylate and activate the JAK/STAT3 pathway [35]. In this study, we found that the EFNB2/EPHB4 axis promoted STAT3 phosphorylation, while inhibition of STAT3 significantly reversed the promotion of LDLR expression by the EFNB2/EPHB4 axis. A recent study demonstrated that aberrant elevated LDLR expression significantly promotes the tumor growth of breast cancer [36]. Our data demonstrated that LDLR mediated the role of EFNB2/EPHB4 axis in promoting intracellular cholesterol levels and tumor growth of CRC LM.

Emerging evidence has shown that hypercholesterolemia is associated with the growth of tumor metastases [37]. A high cholesterol diet has been shown to increase the tumor burden of liver metastasis of melanoma cells [38]. Our analysis showed that a high cholesterol diet significantly reduced the overall survival time of mice with CRC LM, while blocking the EFNB2/EPHB4 axis significantly extended the overall survival time of CRC LM mice with a high cholesterol diet.

Despite the above findings. there are still some shortcomings in this article: 1. The sample size of CRC LM is not enough, which will reduce the credibility of clinical analysis; 2. The clinical transformation potential of EFNB2/EPHB4 axis in CRC LM is not completely clear; 3. Other ways of EFNB2/EPHB4 axis regulating LDLR level are unclear. In this study, we found a specific role of EFNB2/EPHB4 axis in the growth of CRC LM, which may provide specific treatment strategies for patients with CRC LM.

## Materials and methods

### Patients enrollments and samples

Thirty cases of CRC tissues, adjacent paired noncancerous tissues, and matched liver metastasis tissues, were collected from the Department of Gastrointestinal Surgery, Renji Hospital, School of Medicine, Shanghai Jiao Tong University. All the CRC patients underwent surgery at the Department of Gastrointestinal Surgery, Renji Hospital, School of Medicine, Shanghai Jiao Tong University between January 2014 and January 2019. Basic information of patients was shown in Table S1. Inclusion and exclusion criteria were showed in supplementary materials and methods.

#### Sample collection

CRC tissues and noncancerous tissues in this study were quickly obtain from the specimen by surgical excision when the surgery was over. CRC tissue was obtained from the tumor area without apparent necrosis and noncancerous tissues was obtained 5 cm away from the tumor margin. All the tissue were separately loaded into the EP tubes and frozen in liquid nitrogen.

### Cell experiments

#### Reagents and inhibitors

Cholesterol was purchased from Sigma-Aldrich (St. Louis, MO, USA) (C3045) and dissolved in anhydrous ethanol for cell experiments. NVP-BHG712 (50 mg) was purchased from Selleck (Selleckchem, Houston, TX, USA) and dissolved in DMSO. A concentration of 25 nM was used in cell experiments, and mice were orally treated with 3 mg/kg. SH-4-54 was purchased from Selleck and dissolved in DMSO, and 150 nM solutions were used in cell experiments.

#### Cell culture

CRC cell lines used in this article, including SW620, LoVo, SW480, HT29, RKO, and HCT-116 (human CRC cell lines) cells and NCM460 cells were obtained and identified by the Cell Bank of the Chinese Academy of Sciences (Shanghai, China). All cell lines were cultured in Dulbecco’s modified Eagle’s medium (DMEM) supplemented with 10% fetal bovine serum (FBS) and 1% penicillin and streptomycin. Cell culture condition was 37 °C with a 5% CO_2_-humidified atmosphere.

#### Small-interfering RNA (siRNA) transfection

The siRNAs for EPHB1, EPHB2, EPHB3, EPHB4, EPHB5, EPHB6, and EPHA4 were purchased from Shanghai GenePharma Co., Ltd. (Shanghai, China). Their sequences are shown in Table S2, and experimental methods were performed as previously described [39]. Experimental procedures are shown in Supplementary materials and methods.

#### Lentivirus transfection

Full length human EFNB2 cDNA was transfected into CRC cell lines using a lentivirus to generate Lentivirus-EFNB2 (EFNB2-OE). A plasmid with ectopic expression of a C-terminal truncated form of EFNB2 (ΔC EFNB2 and ΔC + H EFNB2) and an N-terminal truncated form of EFNB2 (ΔE EFNB2) was established. Lentivirus-NC was used as the negative control (vector). One short-hairpin RNA (shRNA) sequence against EFNB2 was transfected into CRC cell lines to generate sh-EFNB2, while sh-NC was used as the negative control. The sequences are shown in Table S2. All the lentivirus-EFNB2 cDNAs were purchased from Shanghai GenePharma Co., Ltd (Shanghai, China).

#### Real-time quantitative polymerase chain reaction (RT-qPCR)

Trizol was used to extract RNA, and total RNA was reverse transcribed to cDNA using PrimeScript^TM^ Kits (Takara Bio Inc., Shiga, Japan). 18S RNA was used as an internal control. The sequences of the primers used are shown in Table S2. The relative expression of the target gene was calculated by the ^−△^Ct or ^−△△^Ct method.

#### Western blotting

Total protein was extracted and protein concentration was detected by BCA assay. Western blot analysis was performed as previously described [39]. EFNB2 (ab69858, Abcam, Cambridge, UK), EPHB4 (ab150545, ab98933, Abcam), LDLR (ab52818, Abcam), β-catenin (ab32572, Abcam), VLDLR (ab203271, Abcam), SCARB1(ab52629, Abcam), STAT3(ab68153, Abcam), p-STAT3(ab267373, Abcam), JAK2(ab108596, Abcam), p-JAK2(ab108596, Abcam), SREBP2(ab30682, Abcam), proliferating cell nuclear antigen (PCNA; Proteintech Group, Inc., Sankt Leon-Rot, Germany) primary antibodies were used. Horseradish peroxidase (HRP)-conjugated Affinipure Goat Anti-Rabbit IgG (H + L) (SA00001-2) and HRP-conjugated Affinipure Goat Anti-Mouse IgG (H + L) (SA00001-1) were obtained from Proteintech Group, Inc (Chicago, US).

#### Immunohistochemistry

All tissues were paraffin-embedded and cut into 4 μm thick sections. All sections were dewaxed with xylene and hydrated with alcohol. Sodium citrate was used for antigen retrieval, and 0.3% hydrogen peroxide (H_2_O_2_) was used to block endogenous peroxidase. After blocking non-specific sites with bovine serum albumin, all the sections were incubated with an appropriate primary antibody and secondary antibody. We used 3,3′-diaminobenzidine (DAB) kits (ab64238, Abcam) for visualization, and hematoxylin was used to stain the nuclei. All sections were dehydrated with alcohol and sealed with neutral resin. The immunohistochemistry (IHC) staining score was calculated based on pixel intensity, as follows: no staining, 1; weak staining, 2; moderate staining, 3; and strong staining, 4.

#### Cholesterol detection

The cholesterol levels in the CRC cells and liver metastatic tissue of CRC were detected using Cholesterol/Cholesteryl Ester Quantitation Assay kits (ab65359, Abcam). An amount of 10^6^ CRC cells or 10 mg liver metastatic tissue of CRC were used to detect the cholesterol level.

#### Luciferase reporter assay

The STAT3 overexpressed plasmids were transfected into CRC cells using Roche X-tremeGENE HP DNA Transfection Reagent (Roche Diagnostics, Basel, Switzerland). The luciferase plasmid of each group was added separately. CRC cells were co-cultured with Dual-Glo Luciferase Reagent. The fluorescence values were detected using Dual-Glo Luciferase Assays of SpectraMax M5.

#### ChIP PCR assay

ChIP PCR assays were conducted using Pierce™ Agarose ChIP Kits (26156, Thermo Fisher Scientific, Waltham, MA, USA). CRC cells were fixed with 1% formaldehyde and glycine. The cell chromosomes were fragmented using MNase Digestion Buffer Working Solution and Micrococcal Nuclease. A chromosomal solution of each group was incubated with STAT3 antibody, IgG4 (negative control) and RNA polymerase II antibody (positive control). The immune complex was precipitated and washed, and the DNA samples were recovered. The ChIP results were validated using PCR assays.

### In vivo modeling

All mice were randomly divided into groups. Blindness is used in animal experiments. All animal experiments were approved by the Research Ethics Committee of Renji Hospital and adhered to the local or national requirements for the care and use of laboratory animals. Experimental procedures were showed in supplementary materials and methods.

### LM redigestion and LM cell reculture

LM tissues were obtained from a liver metastasis model established by splenic injection and cut into 2 mm slices. All the LM tissues were treated with Collagenase/hyaluronidase and DNase I. After incubation at 37 °C for 30 min, the incubating mixture was filtered using cell strainers, and the LM cells collected. The LM cells were cultured in DMEM supplemented with 10% FBS and 1% penicillin and streptomycin.

### Statistical analysis

Measurement data are presented as the mean ± standard deviation (SD). SPSS 20.0 (Chicago, IL, USA) and GraphPad Prism 7 software (GraphPad Software, San Diego, CA, USA, www.graphpad.com) were used to conduct the statistical analyses. The correlation of EFNB2 expression with the values of categorical clinical variables in patients with CRC was evaluated using chi-square analysis or Student’s *t*-tests. Measurement data, such as age and tumor size, were evaluated using Student’s *t*-tests, while categorical variables and ranked data, such as gender, T stage, lymph node invasion, and distant metastasis, were analyzed using chi-square tests. Spearman’s rank correlation was used for the analysis of two-way ordered categorical data. Survival curves were generated using the Kaplan–Meier method, and analyzed using log-rank tests. Statistical significance was accepted at *p* < 0.05.

## Supplementary information

supplementary material

Table S1

Table S2

Figure S1

Figure S2

Figure S3

Figure S4

Figure S5

## Data Availability

Public data used in this work can be acquired from the Gene Expression Omnibus (GEO, http://www.ncbi.nlm.nih.gov/geo/). Transcription factors are predicted from JASPAR dataset (https://jaspar.genereg.net/).
