# Supplementary material for: Adaptive activation of EFNB2/EPHB4 axis promotes post-metastatic growth of colorectal cancer liver metastases by LDLR-mediated cholesterol uptake
Source: Oncogene. 2022 Nov 14;42(2):99–112. doi: 10.1038/s41388-022-02519-z (PMC9816060; doi:10.1038/s41388-022-02519-z)
Supplement: Supplementary file 1 — supplementary material [file 41388_2022_2519_MOESM1_ESM.docx]

**Supplementary figure legends**

Figure S1. EFNB2 knockdown inhibits proliferation ability of CRC cells. A. EFNB2 knockdown by shRNA in SW620 and LoVo cells. B. Cell migration ability of SW620 and LoVo cells transfected with sh-EFNB2 or sh-NC. C. PCNA expression in liver metastasis tissues in the sh-EFNB2 and sh-NC groups. Scale bar: 50μm. D. PCNA expression in tumor tissues of liver injection model in sh-EFNB2 and sh-NC groups. Scale bar: 50μm. All experiments were performed in triplicate. Data are presented as the mean ± SD. Student’s *t*-tests were used for statistical analysis. ns. represents no statistical difference, ***p < 0.001.

Figure S2. EFNB2 forward signaling promotes post-metastatic growth of CRC LM. A. Liver metastasis model constructed by spleen injection with vector, EFNB2 FL, EFNB2 ΔC, EFNB2 ΔC + H, and EFNB2 ΔE SW480^luc^ cells (*n* = 6 per group) and signal strength was calculated. B. EdU positive cells of SW480 and HT29 cells transfected with vector, EFNB2 FL, EFNB2 ΔC, EFNB2 ΔC + H, and EFNB2 ΔE. C. EdU positive cells of SW480 and HT29 cells transfected with vector, EFNB2 ΔE, Vector + rEFNB2 ΔC + H, and EFNB2 ΔE + rEFNB2 ΔC + H. All experiments were performed in triplicate. Measurement data are presented as the mean ± SD. Student’s *t*-tests were used for statistical analysis. ***p < 0.001.

Figure S3. The effect of EPHs on cell proliferation. A. EFNB2 overexpression by lentivirus in SW480 and HT29 cells. B. Viability of SW480 and HT29 cells transfected with vector, EFNB2 OE, or EFNB2 OE + siEPHB1, as analyzed using CCK-8 assays. C. Viability of SW480 and HT29 cells transfected with vector, EFNB2 OE, or EFNB2 OE + siEPHB2, as analyzed using CCK-8 assays. D. Viability of SW480 and HT29 cells transfected with vector, EFNB2 OE, or EFNB2 OE + siEPHB3, as analyzed using CCK-8 assays. E. Viability of SW480 and HT29 cells transfected with vector, EFNB2 OE, or EFNB2 OE + siEPHB6, as analyzed using CCK-8 assays. F. Viability of SW480 and HT29 cells transfected with vector, EFNB2 OE, or EFNB2 OE + siEPHA6, as analyzed using CCK-8 assays. G. Efficiency of interference in EPH mRNA levels by siRNA. H. EdU positive cells of SW480 and HT29 cells transfected with vector, EFNB2-OE, EFNB2-OE + siEPHB4, or EFNB2-OE + NVP-BHG712. I. Liver metastasis model created by spleen injection with vector, EFNB2-OE, EFNB2-OE + siEPHB4, or EFNB2-OE + NVP-BHG712 SW480^luc^ cells (*n* = 6 per group) and signal strength was calculated. J. Liver injection model with vector, EFNB2-OE, EFNB2-OE + siEPHB4, or EFNB2-OE + NVP-BHG712 SW480^luc^ cells (*n* = 6 per group) and signal strength was calculated. All experiments were performed in triplicate. Measurement data are presented as the mean ± SD. Student’s *t*-tests were used for statistical analysis. ***p < 0.001.

Figure S4. Analysis of cholesterol synthesis and uptake related genes in CRC LM. A. Cholesterol levels were detected in tumor tissues of the CRC LM model, including the sh-NC and sh-EFNB2 groups (*n* = 5 per group). B. Expression of HMGCS1 in CRC and LM (GSE6988, GSE35834, and GSE49335). C. Expression of HMGCR in CRC and LM (GSE6988, GSE35834, and GSE49335). D. Expression of MSMO1 in CRC and LM (GSE6988, GSE35834, and GSE49335). E. Expression of DHCR24 in CRC and LM (GSE6988, GSE35834, and GSE49335). F. Expression of LDLR in CRC and LM (GSE6988 and GSE49335). G. Expression of VLDLR in CRC and LM (GSE6988, GSE35834, GSE49335). H. Expression of SCARB1 in CRC and LM (GSE6988, GSE35834, and GSE49335). Measurement data are presented as the mean ± SD. Student’s *t*-tests were used for statistical analysis. ns. indicates no statistical difference; *p < 0.05; **p < 0.01; ***p < 0.001.

Figure S5. The EFNB2/EPHB4 axis promotes LDLR expression in CRC LM. A. Correlation of EFNB2 and LDLR, VLDLR, and SCARB1 expression in CRC LM (GSE6988). B. Correlation of EFNB2 and HMGCS1, HMGCR, MSMO1, DHCR24, and NSDHL expression in CRC LM (GSE6988). C. Expression of cholesterol uptake related genes (LDLR, VLDLR, and SCARB1), and cholesterol synthesis related genes (HMGCS1, HMGCR, NSDHL, MSMO1, and DHCR24) in CRC LM tumor tissues, including the sh-NC and sh-EFNB2 groups (*n* = 5 per group). D. Expression of cholesterol uptake related genes (LDLR, VLDLR, and SCARB1), and cholesterol synthesis related genes (HMGCS1, HMGCR, NSDHL, MSMO1, and DHCR24) in HT-29 and LoVo cells in FBS-free culture. All experiments were performed in triplicate. Measurement data are presented as the mean ± SD. Student’s *t*-tests were used for statistical analysis. ns. indicates no statistical difference; ***p < 0.001.

Table S1 Basic information of patients' enrollments

Table S2 primers used in this study

**Supplementary materials and methods**

**Patients enrollments and samples**

Inclusion and exclusion criteria were follows:

Inclusion criteria: Inclusion criteria: 1. Male or female, age ≥18 years; 2. CRC was diagnosed by colonoscopy + pathological biopsy, and LM was diagnosed by enhanced abdominal CT and MRI before surgery; 3. No distant metastasis to other organs; 4. No liver or renal dysfunction or other contraindications; 5. Subject can be surgically evaluated for radical surgery (simultaneous removal of primary and metastatic tumor); 6. Subject have no other serious diseases that conflict with this study; 7. The subject agrees to participate in this study and has signed the informed consent.

Exclusion criteria: 1. CRC patient who have received neoadjuvant chemotherapy; 2. Patient undergoing secondary surgery for colorectal cancer; 3. Have recently undergone major surgery or any surgery over 2 hours; 4. Other complications leading to the impossibility of radical resection; 5. Patient who participated in or are participating in other clinical trials in the 4 weeks prior to enrollment; 6. Patient with serious postoperative complications who cannot receive follow-up diagnosis and treatment; 7. Receiving parenteral or enteral nutrition; 8. Patient cannot participate in this study for other reasons; 9. The subject did not comply with the study or failed to follow the follow-up procedure; 10. The subject has a medical condition that would interfere with the conduct of the study, such as mental illness or substance abuse disorder.

**Small-interfering RNA (siRNA) transfection:**

Belief, an appropriate amount of colorectal cancer cells was added to 6-well plates for culture, and the cells in each well grew to about 60% for siRNA transfection. Two sterile EP tubes were taken, one was added with 250ul OPTI-MEM medium and 5ul RNAiMAX. The other was supplemented with 250ul OPTI-MEM medium and 5ul small interference fragments. Then, the mixture was added to each well of the 6-well plate, and 1ml of 10%FBS DMEM medium without double antibodies was added. After 48 hours, the interference efficiency was detected.

**In vivo modeling**

BALB/C null mice (male, 4 weeks) were used in this study. To generate a liver metastasis model, all nude mice were anesthetized with 0.5% pentobarbital. The abdominal cavity was opened and 1x10^6 cells/null mouse were injected into the spleen. After the mice were sacrificed, the liver metastasis tissues were excised. All tissues were fixed with 4% paraformaldehyde. For the generation of an orthotopic model of CRC, all nude mice were anesthetized with 0.5% pentobarbital. After opening the abdominal cavity, 1x10^6 cells/null mouse were injected into the ileocecum. After the mice were sacrificed, the tumor tissues were excised. All tissues were fixed with 4% paraformaldehyde. To generate a model using hepatic injection of CRC cells, 5x10^6 cells/null mouse were injected into the liver. To generate a model of pulmonary metastasis, 5x10^6 cells/null mouse were injected into tail vein. To generate a model of high cholesterol, nude mice were fed with a high cholesterol diet (TP0504, Nantong Trophic Feed Technology Co., LTD, China).
