# Supplementary material for: Adaptive activation of EFNB2/EPHB4 axis promotes post-metastatic growth of colorectal cancer liver metastases by LDLR-mediated cholesterol uptake
Source: Oncogene. 2022 Nov 14;42(2):99–112. doi: 10.1038/s41388-022-02519-z (PMC9816060; doi:10.1038/s41388-022-02519-z)
Supplement: Supplementary file 2 — Table S1 [file 41388_2022_2519_MOESM2_ESM.docx]

Table S1 Basic information of patients' enrollments

|  |  | **EFNB2 in LM** | | | | ***P value*** | |
| --- | --- | --- | --- | --- | --- | --- | --- |
|  |  | **Low**（n=15） | | **High(n=15)** | |  |  |
| **Age (year)** | | 65.67±4.72 | | 68.77±4.14 | | 0.509 | |
| **Sex** | |  | |  | |  | |
| Male | | 9 | | 10 | | 0.705 | |
| Female | | 6 | | 5 | |  |  |
| **Tumor size (cm3)** | | 32.85±9.61 | | 29.23±4.889 | | 0.739 | |
| **T stage** | |  |  |  |  |  |  |
| T1 and T2 | | 1 | | 0 | | 1 | |
| T3 and T4 | | 14 | | 15 | |  |  |
| **Lymph node invasion** | |  |  |  |  |  |  |
| positive | | 11 | | 12 | | 0.666 | |
| negative | | 4 | | 3 | |  |  |
| **Cholesterol level in LM(ng/mg)** | | 1576.62±109.21 | | 2358.37±157.56 | | **<0.001** | |
