# Supplementary material for: Adaptive activation of EFNB2/EPHB4 axis promotes post-metastatic growth of colorectal cancer liver metastases by LDLR-mediated cholesterol uptake
Source: Oncogene. 2022 Nov 14;42(2):99–112. doi: 10.1038/s41388-022-02519-z (PMC9816060; doi:10.1038/s41388-022-02519-z)
Supplement: Supplementary file 3 — Table S2 [file 41388_2022_2519_MOESM3_ESM.docx]

Table S2 primers used in this study

|  | | **Sequence** |
| --- | --- | --- |
|  |  |  |
| **EPHB1 siNC** | **Sense** | 5'-GAACAAUUGUAAAGGCACACGAGAA-3' |
|  | **Antisense** | 5'-UUCUCGUGUGCCUUUACAAUUGUUC-3' |
| **EPHB1 si1** | **Sense** | 5'-GAAGAAACGUUAAUGGACACCAGAA-3' |
|  | **Antisense** | 5'-UUCUGGUGUCCAUUAACGUUUCUUC-3' |
| **EPHB1 si2** | **Sense** | 5'-CAGAGGAAGUGGACGUGCCCAUCAA-3' |
|  | **Antisense** | 5'-UUGAUGGGCACGUCCACUUCCUCUG-3' |
| **EPHB1 si3** | **Sense** | 5'-CCAUCUGCACCUGUCGGACCGGUUA-3' |
|  | **Antisense** | 5'-UAACCGGUCCGACAGGUGCAGAUGG-3' |
| **EPHB2 siNC** | **Sense** | 5'-CGGGAGGUGUAGAUGCCCUAAACAA-3' |
|  | **Antisense** | 5'-UUGUUUAGGGCAUCUACACCUCCCG-3' |
| **EPHB2 si1** | **Sense** | 5'-GGGCUGGAUGGUGCAUCCUCCAUCA-3' |
|  | **Antisense** | 5'-UGAUGGAGGAUGCACCAUCCAGCCC-3' |
| **EPHB2 si2** | **Sense** | 5'-CAGAAUGGCGCCAUCUUCCAGGAAA-3' |
|  | **Antisense** | 5'-UUUCCUGGAAGAUGGCGCCAUUCUG-3' |
| **EPHB2 si3** | **Sense** | 5'-CGGAAGAGGUGGAUGUACCCAUCAA-3' |
|  | **Antisense** | 5'-UUGAUGGGUACAUCCACCUCUUCCG-3' |
| **EPHB3 siNC** | **Sense** | 5'-CCCGUGGUAGUCCUCGUAAUAGGAA-3' |
|  | **Antisense** | 5'-UUCCUAUUACGAGGACUACCACGGG-3' |
| **EPHB3 si1** | **Sense** | 5'-GCCAGCAUCUGCACCUGCCACAAUA-3' |
|  | **Antisense** | 5'-UAUUGUGGCAGGUGCAGAUGCUGGC-3' |
| **EPHB3 si2** | **Sense** | 5'-CCCGAGGUGUGAUCUCCAAUGUGAA-3' |
|  | **Antisense** | 5'-UUCACAUUGGAGAUCACACCUCGGG-3' |
| **EPHB3 si3** | **Sense** | 5'-GAUCUCCAAUGUGAAUGAAACCUCA-3' |
|  | **Antisense** | 5'-UGAGGUUUCAUUCACAUUGGAGAUC-3' |
| **EPHB4 siNC** | **Sense** | 5'-AGCCAGUGUAACCGUCACUGGUAUU-3' |
|  | **Antisense** | 5'-AGCCAGUGUAACCGUCACUGGUAUU-3' |
| **EPHB4 si1** | **Sense** | 5'-CCGCAGCUUUGGAAGAGACCCUGCU-3' |
|  | **Antisense** | 5'-AGCAGGGUCUCUUCCAAAGCUGCGG-3' |
| **EPHB4 si2** | **Sense** | 5'-AGCUGACUGUGAACCUGACUCGAUU-3' |
|  | **Antisense** | 5'-AAUCGAGUCAGGUUCACAGUCAGCU-3' |
| **EPHB4 si3** | **Sense** | 5'-CCAUGCCCAGCCAAUAGCCACUCUA-3' |
|  | **Antisense** | 5'-UAGAGUGGCUAUUGGCUGGGCAUGG-3' |
| **EPHB6 siNC** | **Sense** | 5'-AGGUGCGACGACCGUUUGUUGACUU-3' |
|  | **Antisense** | 5'-AAGUCAACAAACGGUCGUCGCACCU-3' |
| **EPHB6 si1** | **Sense** | 5'-CAUCUGAGAUUGGCUGGCUCACCUA-3' |
|  | **Antisense** | 5'-UAGGUGAGCCAGCCAAUCUCAGAUG-3' |
| **EPHB6 si2** | **Sense** | 5'-CGGGCACACGUACCCUACAUCUUAG-3' |
|  | **Antisense** | 5'-CUAAGAUGUAGGGUACGUGUGCCCG-3' |
| **EPHB6 si3** | **Sense** | 5'-CAUCUUAGAGGUGCAGGCUGUUAAU-3' |
|  | **Antisense** | 5'-AUUAACAGCCUGCACCUCUAAGAUG-3' |
| **EPHA4 siNC** | **Sense** | 5'-GCGAAGUAUUGUUCCGUUAGAAAUU-3' |
|  | **Antisense** | 5'-AAUUUCUAACGGAACAAUACUUCGC-3' |
| **EPHA4 si1** | **Sense** | 5'-GCGAAUGAAGUUACCUUAUUGGAUU-3' |
|  | **Antisense** | 5'-AAUCCAAUAAGGUAACUUCAUUCGC-3' |
| **EPHA4 si2** | **Sense** | 5'-CAGACAACGACAAAGAGCGUUUCAU-3' |
|  | **Antisense** | 5'-AUGAAACGCUCUUUGUCGUUGUCUG-3' |
| **EPHA4 si3** | **Sense** | 5'-CAGAAUACAGGUGGCCGCCAGGACA-3' |
|  | **Antisense** | 5'-UGUCCUGGCGGCCACCUGUAUUCUG-3' |
| **LDLR siNC** | **Sense** | 5'-GACAAGGGUAUGUCAGAACGGCACA-3' |
|  | **Antisense** | 5'-UGUGCCGUUCUGACAUACCCUUGUC-3' |
| **LDLR si1** | **Sense** | 5'-CAGUGGGCGACAGAUGCGAAAGAAA-3' |
|  | **Antisense** | 5'-UUUCUUUCGCAUCUGUCGCCCACUG-3' |
| **LDLR si2** | **Sense** | 5'-ACGUGCUUGUCUGUCACCUGCAAAU-3' |
|  | **Antisense** | 5'-AUUUGCAGGUGACAGACAAGCACGU-3' |
| **LDLR si3** | **Sense** | 5'-GACCGGGAAUAUGACUGCAAGGACA-3' |
|  | **Antisense** | 5'-UGUCCUUGCAGUCAUAUUCCCGGUC-3' |
| **STAT3 siNC** | **Sense** | 5'-UGGUAACAAGGGACUCAUCACCGCA-3' |
|  | **Antisense** | 5'-UGCGGUGAUGAGUCCCUUGUUACCA-3' |
| **STAT3 si1** | **Sense** | 5'-UGGCCCAAUGGAAUCAGCUACAGCA-3' |
|  | **Antisense** | 5'-UGCUGUAGCUGAUUCCAUUGGGCCA-3' |
| **STAT3 si2** | **Sense** | 5'-CAGCUCUACAGUGACAGCUUCCCAA-3' |
|  | **Antisense** | 5'-UUGGGAAGCUGUCACUGUAGAGCUG-3' |
| **STAT3 si3** | **Sense** | 5'-CACAUGCCACUUUGGUGUUUCAUAA-3' |
|  | **Antisense** | 5'-UUAUGAAACACCAAAGUGGCAUGUG-3' |
| **EFNB2 shNC** | **Sense** | 5'-TTCTCCGAACGTGTCACGT-3' |
|  |  |  |
| **EFNB2 sh1** | **Sense** | 5'-GCAGAACTGCGATTTCCAAAT-3' |
|  |  |  |
| **EFNB2 sh2** | **Sense** | 5'-GAGCCTATCTATTGGAATTCC-3' |
|  |  |  |
| **EFNB2 sh3** | **Sense** | 5'-GGAATTCCTCGAACTCCAAAT-3' |
|  |  |  |
| **EFNB2** | **Forward** | 5’-GAATTCAGCCCTAACCTCTGG-3’ |
|  | **Reverse** | 5’-ATCTTCATGGCTCTTGTCTGG-3’ |
| **18S** | **Forward** | 5’-TGCGAGTACTCAACACCAACA-3’ |
|  | **Reverse** | 5’-GCATATCTTCGGCCCACA-3’ |
| **EPHB1** | **Forward** | 5’-CAAGTTCAGTGGCAAGATGTG-3’ |
|  | **Reverse** | 5’-GCTACAGACGATAGAGATGGC-3’ |
| **EPHB2** | **Forward** | 5’-CTCATTGCTGTGGTTGTCATC-3’ |
|  | **Reverse** | 5’- CTCGTAGGTGAAAGGATCGATG-3’ |
| **EPHB3** | **Forward** | 5’-GTGGTCATCGCTATCGTCTG-3’ |
|  | **Reverse** | 5’-CTCCCGAACAGCCTCATTAG-3’ |
| **EPHB4** | **Forward** | 5’-TTGTGATGTGGGAGGTGATG-3’ |
|  | **Reverse** | 5’-CCGGTAGTCCTGTTCAATGG-3’ |
| **EPHB6** | **Forward** | 5’-TTGTGTCCTCGAATGGCAG-3’ |
|  | **Reverse** | 5’-GACTCAGGGTGTACAGCTAAG-3’ |
| **EPHA4** | **Forward** | 5’-GGAGAACTTGGGTGGATAGC-3’ |
|  | **Reverse** | 5’-TTGGTAGGTTCGGATTGGTG-3’ |
| **LDLR** | **Forward** | 5’-ACGGTGGAGATAGTGACAATG-3’ |
|  | **Reverse** | 5’-CAGGCAAAGGAAGACGAGG-3’ |
| **VLDLR** | **Forward** | 5’-CTGGGTATGCGACGATGATG-3’ |
|  | **Reverse** | 5’-CTTGGTGTGTATGACTGGCTG-3’ |
| **SCARB1** | **Forward** | 5’-AATAAGCCCATGACCCTGAAGC-3’ |
|  | **Reverse** | 5’-GCCCCACATGATCTCACCC-3’ |
| **HMGCS1** | **Forward** | 5’-GGATGAAGGAGTAGGACTTGTG-3’ |
|  | **Reverse** | 5’-GGGAGTCTTGGTACTTTCTTGG-3’ |
| **HMGCR** | **Forward** | 5’-ACAGATACTTGGGAATGCAGAG-3’ |
|  | **Reverse** | 5’- CTGTCGGCGAATAGATACACC-3’ |
| **MSMO1** | **Forward** | 5’- TGAACTTCATTGGAAACTATGCTTC-3’ |
|  | **Reverse** | 5’- TCTTTCAGGAAGGTTTACGTGAG-3’ |
| **NSDHL** | **Forward** | 5’-AGGGCGTCGATATCAAGAATG-3’ |
|  | **Reverse** | 5’-CGGATGGCTGTGGTTAAGAA-3’ |
| **DHCR24** | **Forward** | 5’-GTGAAACACTTTGAAGCCAGG-3’ |
|  | **Reverse** | 5’-AGCCATCAAACATCTCCCAG-3’ |
