# Supplementary figures and images for: Adaptive activation of EFNB2/EPHB4 axis promotes post-metastatic growth of colorectal cancer liver metastases by LDLR-mediated cholesterol uptake
Source: Oncogene. 2022 Nov 14;42(2):99–112. doi: 10.1038/s41388-022-02519-z (PMC9816060; doi:10.1038/s41388-022-02519-z)

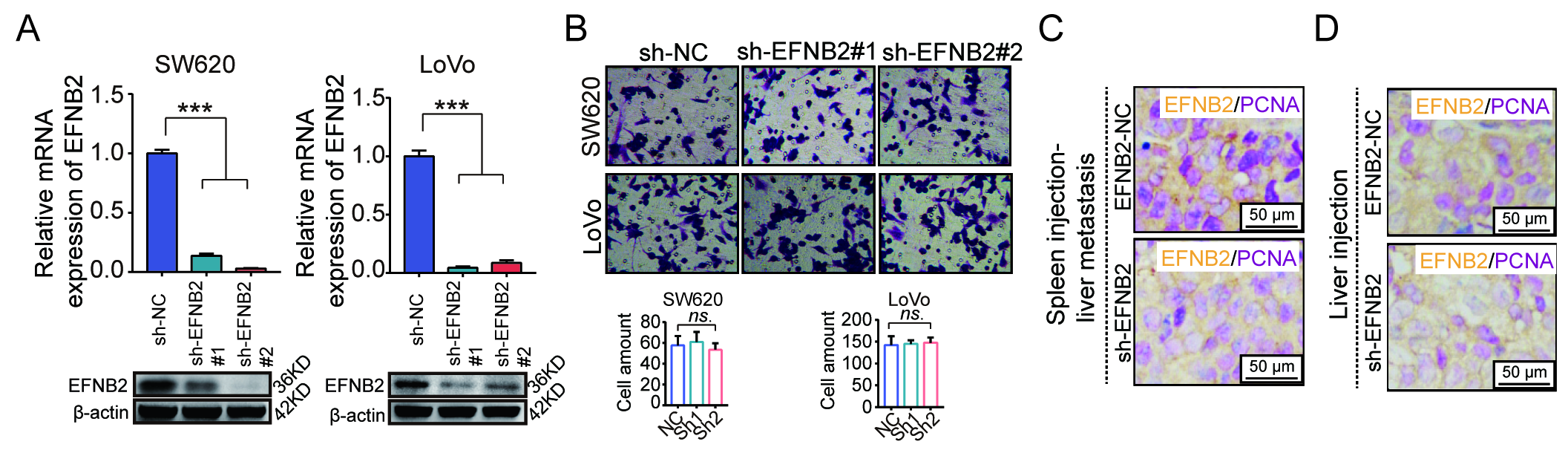

Supplement: Supplementary file 4 — Figure S1 [file 41388_2022_2519_MOESM4_ESM.tif]

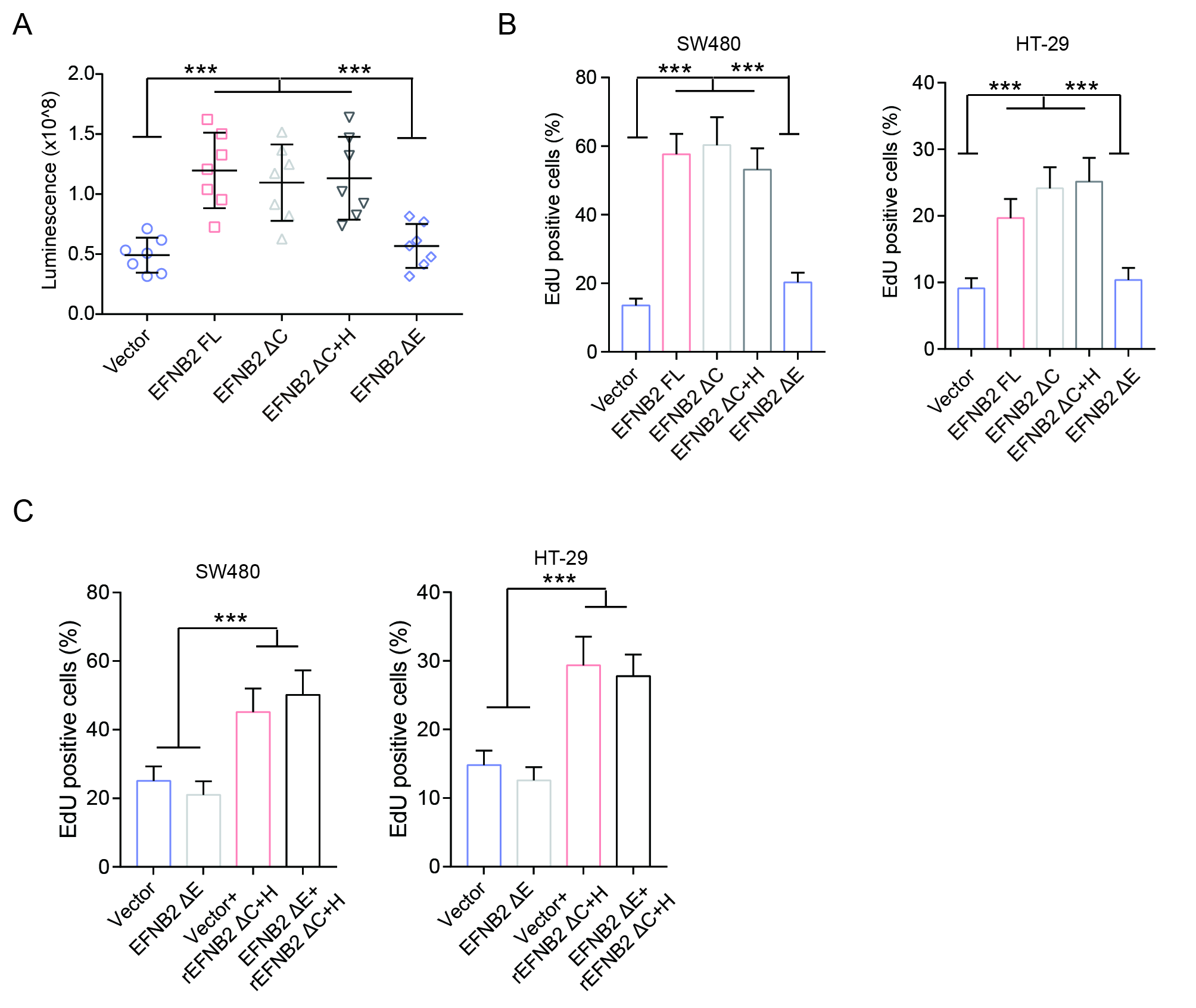

Supplement: Supplementary file 5 — Figure S2 [file 41388_2022_2519_MOESM5_ESM.tif]

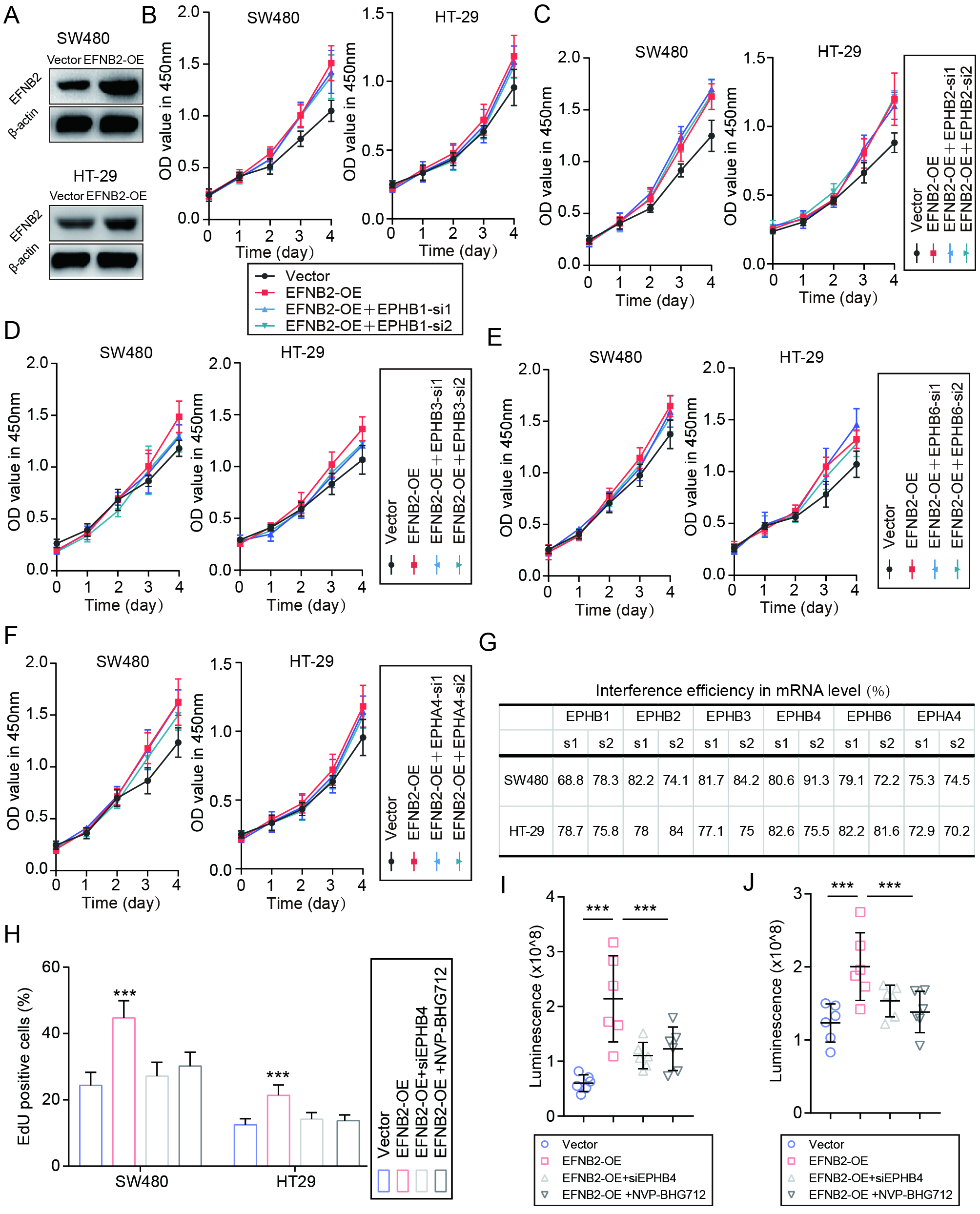

Supplement: Supplementary file 6 — Figure S3 [file 41388_2022_2519_MOESM6_ESM.tif]

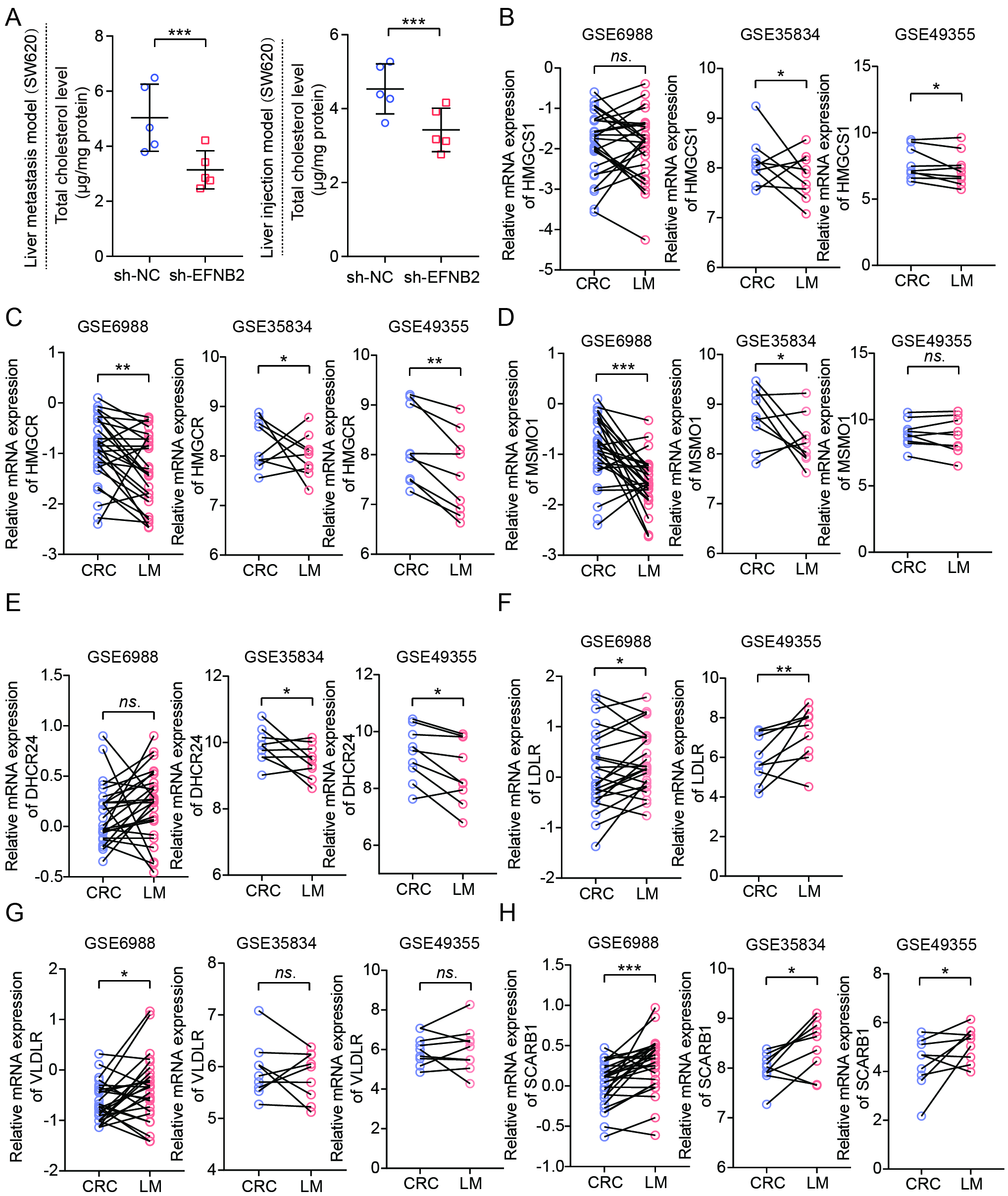

Supplement: Supplementary file 7 — Figure S4 [file 41388_2022_2519_MOESM7_ESM.tif]

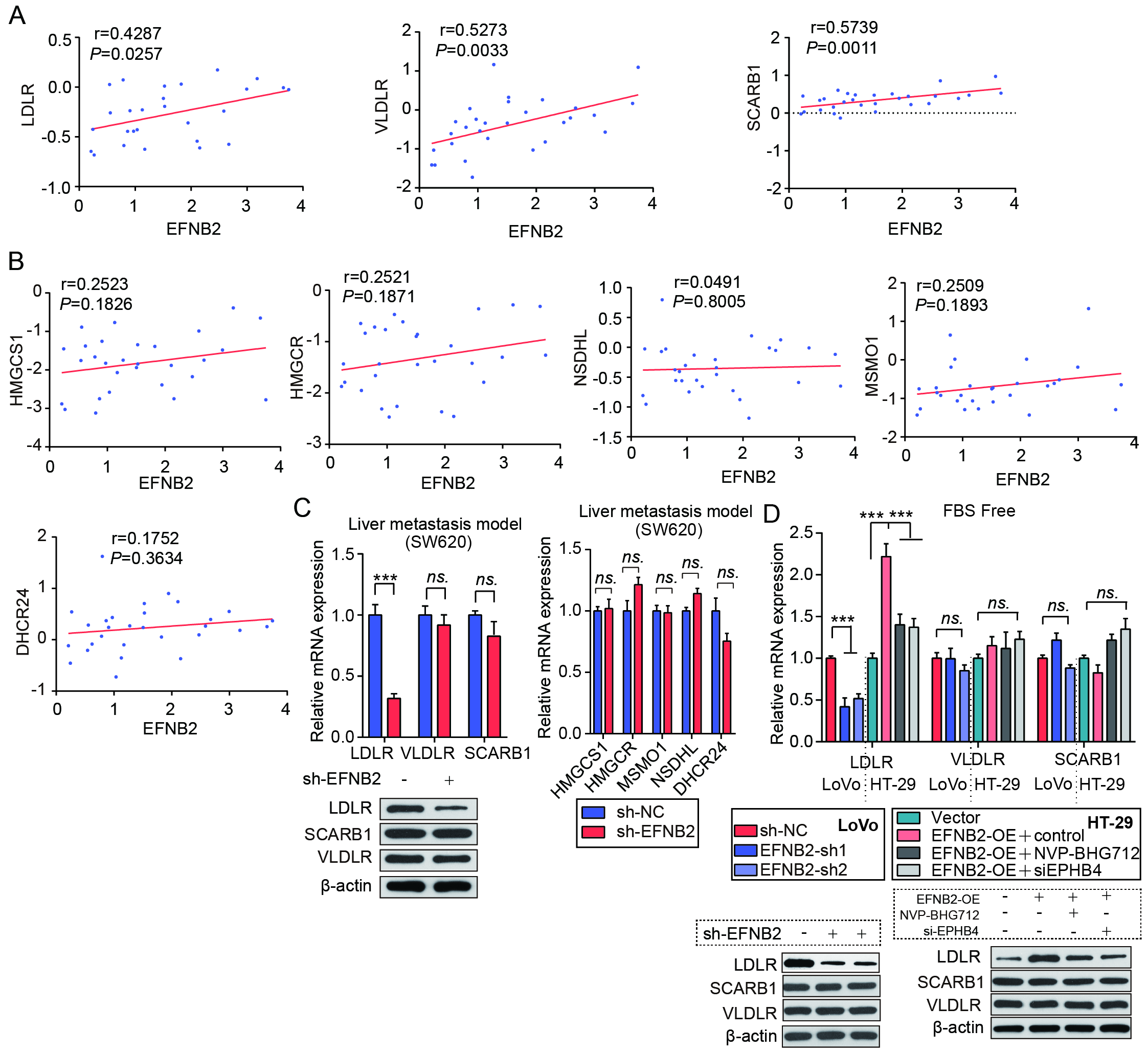

Supplement: Supplementary file 8 — Figure S5 [file 41388_2022_2519_MOESM8_ESM.tif]
